# Induction of site-specific chromosomal translocations in embryonic stem cells by CRISPR/Cas9

**DOI:** 10.1038/srep21918

**Published:** 2016-02-22

**Authors:** Junfeng Jiang, Li Zhang, Xingliang Zhou, Xi Chen, Guanyi Huang, Fengsheng Li, Ruizhe Wang, Nancy Wu, Youzhen Yan, Chang Tong, Sankalp Srivastava, Yue Wang, Houqi Liu, Qi-Long Ying

**Affiliations:** 1 Research Center of Developmental Biology, Histology and Embryology Department, Second Military Medical University, Shanghai, 200433, China; 2 Eli and Edythe Broad Center for Regenerative Medicine and Stem Cell Research at USC, Department of Stem Cell Biology & Regenerative Medicine, Keck School of Medicine, University of Southern California, Los Angeles, CA, USA; 3 Translational medicine center, Second Military Medical University, Shanghai, 200433, China

## Abstract

Chromosomal translocation is the most common form of chromosomal abnormality and is often associated with congenital genetic disorders, infertility, and cancers. The lack of cellular and animal models for chromosomal translocations, however, has hampered our ability to understand the underlying disease mechanisms and to develop new therapies. Here, we show that site-specific chromosomal translocations can be generated in mouse embryonic stem cells (mESCs) via CRISPR/Cas9. Mouse ESCs carrying translocated chromosomes can be isolated and expanded to establish stable cell lines. Furthermore, chimeric mice can be generated by injecting these mESCs into host blastocysts. The establishment of ESC-based cellular and animal models of chromosomal translocation by CRISPR/Cas9 provides a powerful platform for understanding the effect of chromosomal translocation and for the development of new therapeutic strategies.

Chromosomal translocation, the rearrangement of parts between non-homologous chromosomes, is the most common form of chromosomal abnormality, occurring in one in approximately 500 live births in humans^1^. Chromosomal translocation is caused by double-strand breaks (DSBs) in chromosomes and subsequent non-homologous end joining (NHEJ) between different chromosome arms^2^. Chromosomal translocations that arise during gametogenesis will transmit to all somatic cells of the offspring and might cause heritable disorders, such as infertility^3^, Down’s Syndrome^4^,^5^, schizophrenia^6^, and so on. Chromosomal translocations can also occur in a subset of somatic cells during mitosis, which may be found in various kinds of cancers^7^,^8^,^9^. In addition, new disease cases with previously unobserved chromosomal translocations keep emerging and pose a severe threat to patients’ health^10^,^11^,^12^,^13^,^14^. Mechanistic and functional studies of chromosomal translocations and the development of new therapies, however, have been hampered due to the difficulty in creating cellular or animal models that faithfully recapitulate the chromosomal translocation events.

Mouse embryonic stem cells (mESCs) are derived from the inner cell mass (ICM) of a mouse blastocyst and have the ability to self-renew and differentiate into all types of cells in the body^15^,^16^. If site-specific chromosomal translocations can be induced in mESCs by the CRISPR/Cas9 system, a mESC model, or a mESC-derived cellular model carrying a chromosomal translocation will be achieved. These models will benefit chromosomal translocation research, especially chromosomal translocation research on those cells that are difficult to isolate or proliferate *in vitro*. Additionally, the mESC cell line carrying a chromosomal translocation could be labeled and microinjected into blastocysts to generate mouse chimeras as a mouse model of subjects in which only a subset of cells carry the chromosomal translocation, or eventually a mouse model in which every cell carries the chromosomal translocation, as illustrated in the schematic diagram (Fig. 1a).

Recent advances in genome editing technologies, especially the development of CRISPR/Cas9 system have made it possible to generate site-specific chromosomal editing in mammals^17^,^18^,^19^,^20^,^21^. In this system, the Cas9 nuclease and specifically designed single-guide RNAs (sgRNAs), which contain a 20-nucleotides sequence for pairing with target DNA, are artificially co-expressed in cells. sgRNA and Cas9 form an sgRNA/Cas9 effector complex, which induces a double-strand break (DSB) at the intended genomic location (Fig. 1b). Considering that DSBs in nonhomologous chromosomes can lead to chromosomal translocation, we speculated that site-specific chromosomal translocation could be induced by CRISPR/Cas9.

## Results

### Generation of mESCs carrying chromosomal translocations by CRISPR/Cas9

**Figure 1:**
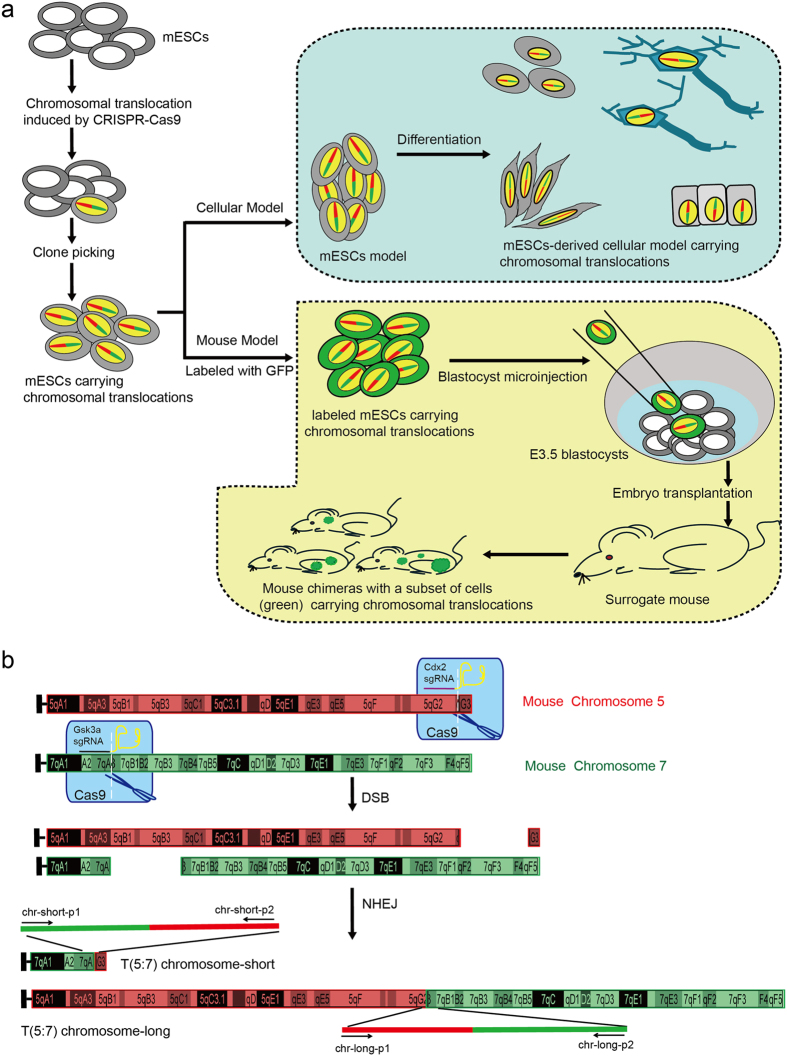
Strategy for generating cellular and mouse models of chromosomal translocation via the ESC- and CRISPR/Cas9-based technologies. (**a**) Strategy for generating mESC models, or mESC-derived cellular models, and mouse models carrying a chromosomal translocation. (**b**) Strategy for generating site-specific chromosomal translocations in mESCs using the CRISPR/Cas9 system. Cdx2 and Gsk3α sgRNAs will guide Cas9 (blue) onto the indicated target sites located in mouse chromosome 5 (red) and chromosome 7 (green), respectively. DSBs will then be induced in these two sites. By activating NHEJ, DSBs can be repaired and the chromosomal translocation T (5:7) may occur in the designated location, thus generating two translocated chromosomes. To show the precise location and the relative length of the chromosomes, the chromosome graphs from the University of California Santa Cruz (UCSC) Genome Browser were used. Primer chr-short-p1 was designed to anneal to chromosome 7 at the site upstream of the predicted DSB point. Primer chr-short-p2 was designed to anneal downstream of the chromosome 5 DSB point. The size of PCR product is expected to be approximately 930 bp if the translocation occurs. Similarly, primers chr-long-p1 and chr-long-p2 were designed to detect T (5:7) chromosome-long, and the size of the PCR product is approximately 300 bp.

**Figure 2:**
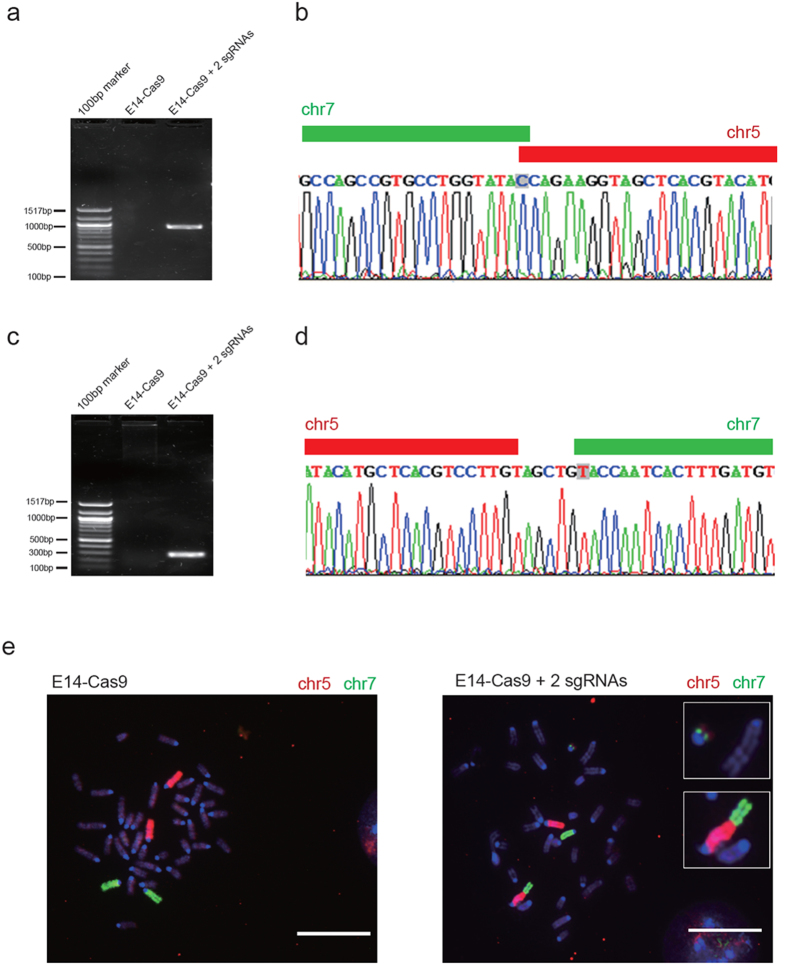
Translocation between chromosome 5 and chromosome 7 mediated by the CRISPR/Cas9. (**a**) PCR analysis with chr-short-p1 and chr-short-p2 primers showing the presence of a ~930 bp PCR product in E14-Cas9 mESCs infected with Cdx2 and Gsk3α-sgRNAs. (**b**) Sequence of the PCR product (in one pMD18-T clone) of the predicted T (5:7) chromosome-short, and one cytosine nucleotide was deleted at the junction point. (**c**) PCR analysis with chr-long-p1 and chr-long-p2 primers showing the presence of a ~300 bp PCR product in E14-Cas9 mESCs infected with Cdx2 and Gsk3α sgRNAs. (**d**) Sequencing of the PCR product (in one pMD18-T clone) of the predicted T (5:7) chromosome-long indicates the addition of five nucleotides at the junction point. (**e**) Fluorescent images of the metaphase chromosomes of mESCs labelled with chromosome 5 (red) and 7 (green) specific probes. Insets zoomed in the two translocated chromosomes. Scale bars represent 10 μm.

Our general strategy for generating ESC-based cellular and animal models of chromosomal translocation is illustrated in Fig. 1a. We first generated an E14TG2a mESC line with stable expression of Cas9 (hereinafter, E14-Cas9) by transfection of a Cas9-expressing vector. As a proof-of-principle study, we first chose Cdx2 and Gsk3α loci as our induced chromosomal translocation target sites for the following reasons: 1. Cdx2 and Gsk3α sgRNAs have been proven to work efficiently in mESCs in our previous experiments. 2. Cdx2 and Gsk3α loci localize on chromosomes 5 and 7, respectively (Fig. 1b). 3. Our previous results suggest that disruption of Cdx2 and Gsk3α will not affect ESC self-renewal, which is necessary for obtaining ESC sub-clones. We designed two pairs of sgRNAs to target the *Cdx2* and *Gsk3α* loci (Fig. 1b). DSBs at these two loci induced by Cas9 were expected to generate two translocated chromosomes, which we named T (5:7) chr-short and T (5:7) chr-long (Fig. 1b). E14-Cas9 mESCs were infected with lentiviral vectors expressing Cdx2-sgRNA and Gsk3α-sgRNA. After 72 h of drug selection, we harvested the cells and extracted the genomic DNA for PCR screening to identify chromosomal translocation events. The PCR primers were designed to span the expected junction point between the two translocated chromosome arms (Fig. 1b). For T (5:7) chr-short, we obtained a PCR product with the predicted size of 930 bps (Fig. 2a). Subsequent sequencing confirmed that the 5′-end half and 3′-end half of the PCR product matched with the sequences at chromosome 5 and 7, respectively (Supplementary Fig. 1c). The appearance of double peaks straight after the junction point, together with the 3 nucleotides deletion suggests that the translocation was mediated by NHEJ (Supplementary Fig. 1a). After sub-cloning into a pMD18-T vector, followed by sequencing, we obtained the clear single-peaked sequence for the full length of the PCR product. The sequences around the junction point further prove the NHEJ mediated chromosomal translocation between chromosome 5 and 7 (Fig. 2b and Supplementary Fig. 1b). Similarly, PCR (Fig. 2c) and sequencing (Fig. 2d, and Supplementary Fig. 1d) analysis confirmed the presence of the T (5:7) chr-long (Fig. 1b). Additionally, we were also able to detect indel mutation in untranslocated chromosomes (Supplementary Fig. 1e,f). To further verify the chromosomal translocation events at the cellular level, we adapted the chromosome painting technology to mark chromosome 5 with red fluorescence and chromosome 7 with green^22^. Both T (5:7) chr-long and T (5:7) chr-short were visually detected in mESCs co-expressing Cas9, Cdx*2*-sgRNA, and Gsk3α-sgRNA but not in mESCs expressing Cas9 only (Fig. 2e). Among the 100 cells observed with clearly painted chromosomes, two showed positive chromosome painting architectures indicative of T (5:7) translocation (Supplementary Fig. 2 and Supplementary Table 1).

To generate a mESC cellular model where every cell carries specific chromosomal translocation, we then passaged sgRNA-infected cells into a 96-well plate at the density of approximately 5 cells per well. Of the total 33 wells, the T (5:7) translocation was identified in 3 wells by PCR analysis (Supplementary Fig. 3a), confirming the efficiency observed by chromosome painting. Then, we seeded one well of translocation-positive cells at a single-cell density and picked up 16 individual colonies. PCR analysis and sequencing confirmed that 3 of these showed the T (5:7) translocation (Supplementary Fig. 3b and Supplementary Table 2). Next, one of the T (5:7) translocated colonies was expanded and confirmed by chromosome painting, and showed that every cell carried the T (5:7) translocation (Supplementary Fig. 3c and Supplementary Table 2). Using the same CRISPR/Cas9 approach, we generated HEK293 cells harboring the human chronic myelogenous leukemia (CML)-associated Philadelphia chromosome by inducing chromosomal translocation between chromosomes 9 and 22, which resulted in the creation of the *BCR-ABL1* fusion gene (Supplementary Fig. 4a)^23^. HEK293 is an immortalized cell line derived from human embryonic kidney cells. We also created the *Bcr-Abl1* fusion gene in mESCs by inducing the T (2:10) chromosomal translocation (Supplementary Fig. 4b).

### mESCs carrying the T (5:7) translocation retain the ability to differentiate into all three germ layers

**Figure 3:**
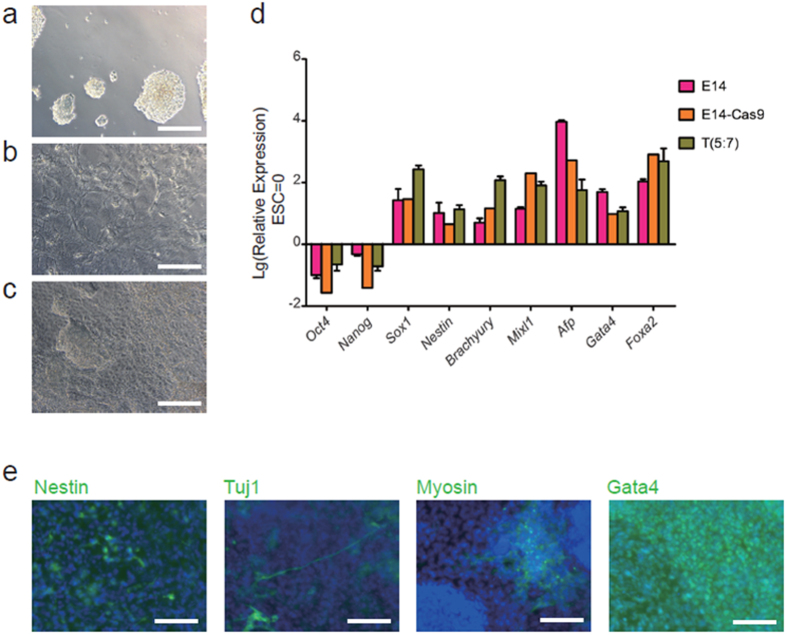
mESCs carrying the T (5:7) translocation retain ability to differentiate into three germ layers. (**a**) T (5:7)-GFP-E14 cells cultured in normal conditions (with LIF). (**b**,**c**) T (5:7)-GFP-E14 cells cultured without LIF for 7 days. (**d**) T (5:7)-GFP-E14 retain the ability to differentiate into all three germ layers *in vitro*. All 3 groups of cells were differentiated for 7 days by removing LIF. The expression levels were analyzed by real-time PCR and compared with undifferentiated cells cultured in the medium with LIF. The expression levels were Lg transformed. Data are represented as the mean ± SD, n = 4. (**e**) Immunofluorescence staining of differentiated T (5:7)-mESCs with anti-Nestin (ectodermal markers), anti-Tuj1 (ectodermal markers), anti-Myosin (mesodermal marker) and anti-Gata4 (endodermal marker) antibodies. Nuclei were stained with DAPI (blue). Scale bars, 200 μm.

To assess whether mESC lines carrying the T (5:7) translocation remain pluripotent, we induced differentiation of these mESCs by removing leukemia inhibitory factor (LIF). Seven days after LIF withdrawal, we found that the cells showed differentiation morphology (Fig. 3a–c), in coincidence with the down-regulation of pluripotency markers *Oct4* and *Nanog* and the up-regulation of the three germ layers’ differentiation-related genes, such as *Sox1, Nestin, Brachyury, Mixl1, Afp, Gata4, Foxa2* (Fig. 3d). Additionally, immunofluorescence staining showed that mESCs carrying the T (5:7) translocation can differentiate into various cell types of the three germ layers (Fig. 3e). Collectively, these results indicate that site-specific chromosomal translocations can be induced in mESCs using the CRISPR/Cas9 system and that mESCs carrying chromosomal translocations remain pluripotent.

### Generation of chimeric mouse embryos from mESCs carrying the T (5:7) chromosomal translocation

**Figure 4:**
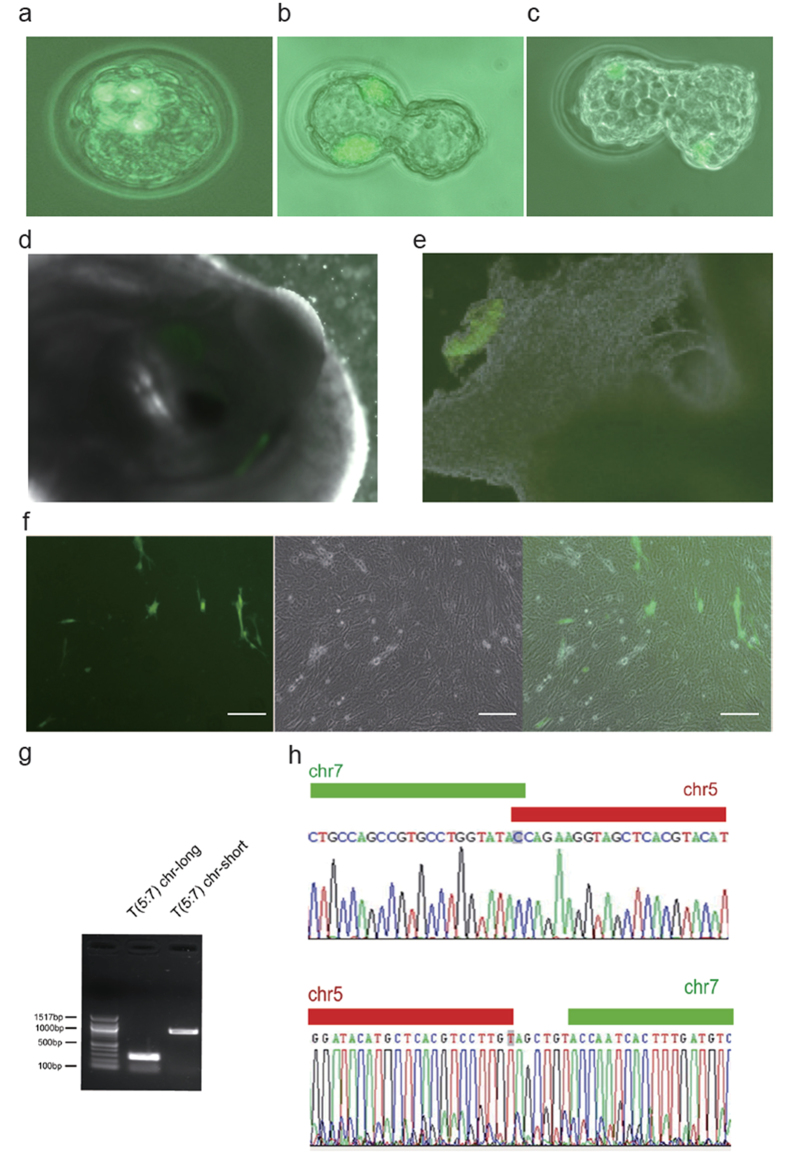
Chimeric mouse embryos carrying the T (5:7) translocation were developed from blastocysts injected with T (5:7)-GFP-E14 cells. (**a**) GFP-positive T (5:7)- mESCs injected into an E3.5 blastocyst. (**b,c**) Injected GFP-positive T (5:7)-mESCs developed and integrated into the receptive embryo one (**b**) or two (**c**) days after injection. (**d**) One representative E13.5 chimeric embryo developed from blastocyst injected with GFP-positiveT (5:7)-mESCs and contained GFP-positive cells. (**e**) GFP-positive tissue was isolated from the chest of an E13.5 chimeric embryo. (**f**) Cultured cells isolated from the hind legs of the chimeric embryos. Some GFP-positive cells shown the contribution of T (5:7)-GFP-E14 cells into the chimeric embryos. Scale bars represent 100 μm. (**g,h**) Both the T (5:7) chromosome-long and the T (5:7) chromosome-short were detected in chimeric mouse embryos by PCR and sequencing analysis.

Next, we tested whether the T (5:7) mESCs could be used to generate chimeric mice carrying specific chromosomal translocations. After being labeled with a Green Fluorescent Protein (GFP) marker, T (5:7) mESCs were injected into B6D2F1 (C57BL/6 and DBA/2 hybrid) mouse blastocysts (Fig. 4a), and the ESC-injected blastocysts were transferred to pseudo-pregnant female C57BL/6 mice. We obtained the E4.5 and E5.5 mouse embryos, and GFP-positive cells were detected at these stages (Fig. 4b,c). From 30 ESC-injected blastocysts, we recovered a total of 12 embryos at E13.5 and detected GFP-positive tissue in one of them (Fig. 4d,e). We also isolated and cultured cells from another 3 embryos in which GFP signal were undetectable *in situ*. One of the embryos gave rise to GFP-positive cells in culture (Fig. 4f). Furthermore, the induced chromosomal translocation could also be detected in the chimeric embryos at genomic DNA level by PCR and sequencing analysis (Fig. 4g,h). Collectively, these results suggest that mESCs carrying chromosomal translocations retain the ability to contribute to chimera formation.

## Discussion

Herein, we have demonstrated the feasibility of creating site-specific chromosomal translocation cellular and animal models via CRISPR/Cas9-mediated genome editing in mESCs. These models provide a valuable platform for the study of chromosomal translocation and the development of new treatments for chromosomal translocation-associated diseases.

Recently, the CRISPR/Cas9 system has been used to successfully induce chromosomal translocations in both cultured HEK293 cell lines and in mice^24^,^25^,^26^. Compared to these reported approaches, our ESC-based approach to generate cellular and animal models of chromosomal translocation has several advantages. By inducing chromosomal translocations in mESCs, we can isolate and expand single mESC harboring specific chromosome translocations. These mESCs can then be used to generate an unlimited amount of nearly any type of cells for the study of how chromosomal translocation affects cellular function. More importantly, this ESC-based strategy could also be used to generate mouse models of different diseases with every cell carrying the same chromosomal translocation, which is impossible to achieve using the somatic cell-based approach. Additionally, mESCs carrying translocated chromosomes are much easier to label and track *in vivo* than in the reported somatic cell-based method.

CRISPR/Cas9-induced chromosomal translocation is a rare event, even in HEK293 cells, which are ideal for transfection with high efficiency^24^,^25^,^26^, mESCs are relatively difficult for transfection and result in low efficiency. To increase chromosomal translocation efficiency in mESCs, we generated mESCs with Cas9 stably integrated and then infected these mESCs with sgRNAs lentivirus to induce chromosomal translocation. By doing this, we were able to achieve ~2% chromosomal translocation efficiency in mESCs. We believe that this efficiency can be further increased by using a gene expression vector containing two sgRNA elements. Currently, another technical hurdle is the low efficiency of establishing cell-lines carrying chromosomal translocation. Our current strategy of picking individual colonies and genotyping one by one is time-consuming. To overcome this hurdle, a new technical improvement that allows monitoring chromosomal translocation events in real-time is needed.

Although the chimeric contribution of T (5:7) mESCs seems to be low, as a proof-of-principle study, we proved chimeric mice can be generated by injecting mESCs carrying induced chromosomal translocation into host blastocysts. The low contribution of ESCs to chimera formation could have been caused by disruption of the Cdx2 gene which was reported to play an important role in development^27^,^28^. We believe that ESCs carrying chromosomal translocations should contribute efficiently to chimera formation if the disrupted genes are not important for embryonic development. Further validation and investment on other diseases-associated chromosomal translocations may be needed in the future.

It has been shown recently, that eukaryotic genomes are highly organized functional architectures with a large number of long-range DNA cis-interactions^29^,^30^,^31^,^32^,^33^,^34^. To what extend a specific chromosome translocation could affect the genome architecture and impact development and other pathological processes are questions worth considering for the future. Moreover, to verify an assumed chromatin interaction, changing the original chromosome structure may be necessary in some cases. Induced chromosomal translocation provides a powerful tool for changing the chromosome structure in ESCs, which may greatly facilitate the study of chromatin interaction.

Multiplex targeting has always been considered to be an advantage of the CRISPR/Cas9 systems^17^,^18^; however, it should be noted that multiplex targeting by Cas9 may lead to unexpected chromosomal translocations when targeting events occur on different chromosome locations. In addition, due to the potential off-target effects of CRISPR/Cas9^35^,^36^, complex chromosomal translocations may arise from off-targeted DSBs and pose a concern for the application of this technology in the future.

## Methods

### Cell culture

Mouse ESCs were cultured on 0.1% gelatin-coated dishes in 5% CO2 at 37 °C. Medium for routine maintenance was GMEM (Sigma, G5414) supplemented with 1% MEM non-essential amino acids (Invitrogen), 10% FCS (HyClone), 0.1 mM β-mercaptoethanol (Invitrogen), 2 mM GlutaMax (Invitrogen), and 100 units/ml LIF (prepared in-house). For serum-free culture, mESCs were maintained in N2B27, supplemented with 3 μM CHIR99021 and 1 μM PD0325901 (Synthesized in the Division of Signal Transduction Therapy, University of Dundee, UK).

### Construction of CRISPR/Cas9 System plasmids

The piggyBac transposon-based vectors were obtained from SBI (PB Transposon vector #PB511B-1, PiggyBac Transposase vector #PB200PA-1). To achieve a higher expression level in ESCs, the CMV-MCS-EF1-puro dual promoter cassette in PB Transposon vector was replaced by a CAG-MCS-IRES-hygro cassette using NheI and ClaI. Human codon optimized Cas9 gene was cloned from hCas9 plasmid (Addgene plasmid 41815) and inserted into the revised piggyBac expression vector with hygromycin resistance. The sgRNAs were designed according to Addgene hCRISPR sgRNA synthesis protocol. Particularly, we screened 23 bp NDA sites containing the NGG sequence at the intended target sites, and then used NCBI BLAST to exclude the sequences that could target any other mouse genomic DNA loci. The intended target sites of Gsk3α and Cdx2 were at the 1^st^ to 2^nd^ exon, and for the Bcr-Abl1 translocation, the intended target sites were at intron to mimic disease related fusion gene formation, as shown in Figure S4. The final target sequences were provided in the Supplementary Information. Sequence of sgRNAs with the U6 promoter + target sequence + guide RNA scaffold + termination signal were synthesized (Integrated DNA Technologies, Inc.). The Gsk3α-sgRNA and Bcr-sgRNA sequence were cloned into pLKO.1-TRC vector that contains a blasticidin resistamce element for lentivirus packaging. The Cdx2-sgRNA and Abl1-sgRNA sequence were cloned into the pLKO.1-TRC vector that contains a puromycin resistance element. PCR products amplified with genomic DNA harvested from cells were sub-cloned into pMD18-T vector (TaKaRa) followed by the instruction of the manufacturer.

### Cell transfection and virus infection

For stable expression of Cas9, 1 × 10^5^ E14TG2a mESCs^37^ were transfected with 2 μg of PB-CAG-hCas9-IRES-hygro and 1 μg PiggyBac Transposase vector using Lipofectamine LTX (Invitrogen), according to the manufacturer’s instructions. The Cas9 stably expressing mESC line, E14-Cas9, was established by adding hygromycin (200 μg/ml) to the medium for 3 weeks after transfection. To package the lentivirus, packaging plasmids psPAX2, VSV-G, and pLKO.1-TRC-based lentiviral vectors that contained the sgRNA sequences were co-transfected into HEK293FT cells using Lipofectamine LTX. After 48 hours, the supernatant was collected and passed through 0.45 μm filters (Millipore). The lentiviral supernatants were added into the culture medium of mESCs in the presence of 8 μg/ml polybrene (Sigma) for the sgRNA expression. After 24 hours, puromycin (1 μg/ml) and blasticidin (5 μg/ml) were added into the cell culture medium for 72 hours to select cells co-expressing the two sgRNAs.

### Chromosomal translocation detection by PCR and sequencing

Cells were harvested and genomic DNA was extracted using the genomic DNA extraction kit (Qiagen). A polymerase Chain Reaction (PCR) was performed by KOD Hot Start DNA Polymerase (EMD) with primers spanning the expected junction point of translocation. The primers used for amplification were the following: chr-short-p1: CAAATCGTGTTTCTGGGGGT, and chr-short-p2: CTGAGGAAATGCCCAGTAAA were used for T (5:7) chromosome-short detection (predicted size: approximately 930 bps). Chr-long-p1: GCCCAGAGCGTTCCCAAGA and chr-long-p2: CGGGTGCGTAGCCATTCCA were used for T (5:7) chromosome-long detection (predicted size: approximately 300 bps). The PCR products were purified and sent for sequencing (Genewiz, Inc.). mBcr-Abl1-P1: TCTCCTGGACTCTACGGCTTC and mBcr-Abl1-P 2: ATTGGTCACTGGCTTTCTTCTG were used for mouse T (2:10) chromosomal translocation (Bcr-Abl1 fusion). hBCR-ABL1-P1: TGTCACCTGCCTCCCTTTCC and hBCR-ABL1-P2: GCTTCACACCATTCCCCATTG were used for human T (9:22) chromosomal translocation (BCR-ABL1 fusion).

### Real-Time PCR Analysis and Immunostaining

Total RNA was extracted using Trizol (Invitrogen). First-strand cDNA was generated using a Quanti-Tect Transcription Kit (Qiagen). The quantitative real-time PCR (RT-PCR) mixtures were prepared using SYBR Green PCR Master Mix and run on an ABI7900HT Fast RT-PCR System (Applied Biosystems, Carlsbad, CA, http://www.lifetechnologies.com/applied-biosystems). RT-PCR was carried out using relative expression levels of pertinent genes that were normalized against GAPDH. Immunostaining was performed according to a standard protocol, using primary antibodies including Nestin (1:100, Santa Cruz), Tuj1 (1:500, Covance, Princeton, NJ, www.covance.com), Myosin (1:100, Developmental Hybridoma Studies Bank) and Gata4 (1:100, Santa Cruz). Alexa Flour fluorescent secondary antibodies (Invitrogen) were used at a dilution of 1:2,000 and nuclei were stained with DAPI (1:5,000).

### Chromosome painting

For chromosome sample preparation, cells were treated with colcemid (0.05 μg/ml) for 2 hours before harvesting the mitotic cells. The cells were treated for 25 minutes at room temperature with hypotonic potassium chloride solution (0.075 M) followed by fixation in acetic acid/methanol (1:3). The mitotic cell suspensions were dropped onto pre-cooled glass slides and air-dried for 24 hours. Fluorescent *in situ* hybridization was performed according to the manufacturer’s protocol using a red fluorescent probe specific for mouse chromosome 5 and a green fluorescent probe for chromosome 7 (Applied Spectral Imaging). Briefly, the chromosome slides were immersed in 2 × SSC and an ethanol series of 70%, 80% and 100% for 2 minutes each. Then, the slides were placed into a 70 °C denaturation solution (70% formamide/2 × SSC, pH7.0) for 4 minutes. Slides were immediately placed, sequentially, in cold 70%, 80% and 100% ethanol (each for 2 min) and air-dried. After denaturation (80 °C for 7 minutes), the probe mixture was added to the denatured chromosome. Hybridization was performed in a humidified dark chamber at 37 °C for 16 hours. After hybridization, the slides were washed in 0.4 × SSC solution at 74 °C for 4 minutes, followed by dipping into washing solution II (4 × SSC/0.1% Tween 20) for 2 minutes. Finally, the slides were stained with DAPI in anti-fade solution. The slides were observed with fluorescence microscope, and the digital images were captured by a CCD camera.

### Chimeric mouse embryos

For the T (5:7) -E14 cells labeling, cells were infected with lentiviral vectors expressing GFP. After drug selection, GFP-positive colonies were picked up, and one clone was used for the following experiments. For production of chimeras with T (5:7)-GFP-E14 cells, embryos were obtained from C57BL/6 female and DBA/2 male crossing. T (5:7)-GFP-E14 cells were trypsinized into single cells and injected into E3.5 blastocysts. Then, the ESC-injected blastocysts were transferred into 2.5 dpc pseudopregnant C57BL/6 females. All mice were purchased from Jackson Labs and all animal procedures were carried out according to IACUC-approved protocols. The mice were housed in a pathogen-free environment. For the embryonic cell cultures, tissues were harvested and trypsinized from E13.5 embryos and cells were cultured in DMEM with 10% FBS at 37 °C in 5% CO2.

## Additional Information

**How to cite this article**: Jiang, J. *et al.* Induction of site-specific chromosomal translocations in embryonic stem cells by CRISPR/Cas9. *Sci. Rep.* **6**, 21918; doi: 10.1038/srep21918 (2016).

## Supplementary Material

Supplementary Information
