# Supplementary material for: Induction of site-specific chromosomal translocations in embryonic stem cells by CRISPR/Cas9
Source: Sci Rep. 2016 Feb 22;6:21918. doi: 10.1038/srep21918 (PMC4761995; doi:10.1038/srep21918)
Supplement: Supplementary Information [file srep21918-s1.pdf]

Supplemental Information

Induction of site-specific chromosome translocations in embryonic stem cells by CRISPR/Cas9

Authors

Junfeng Jiang*[1, 2], Li Zhang*[2, 3], Xingliang Zhou*[2], Xi Chen[2], Guanyi Huang[2], Fengsheng Li[2], Ruizhe Wang[2], Nancy Wu[2], Youzhen Yan[2], Chang Tong[2], Sankalp Srivastava[2], Yue Wang[§, 1], Houqi Liu[§, 1, 3], Qi-Long Ying[§, 2]

Affiliations

1: Research Center of Developmental Biology, Histology and Embryology Department, Second Military Medical University, Shanghai, 200433, China

2: Eli and Edythe Broad Center for Regenerative Medicine and Stem Cell Research at USC, Department of Stem Cell Biology & Regenerative Medicine, Keck School of Medicine, University of Southern California, Los Angeles, CA, USA

3: Translational medicine center, Second Military Medical University, Shanghai, 200433, China

* These authors contribute equally to this work.

§ Corresponding authors

Supplemental figures

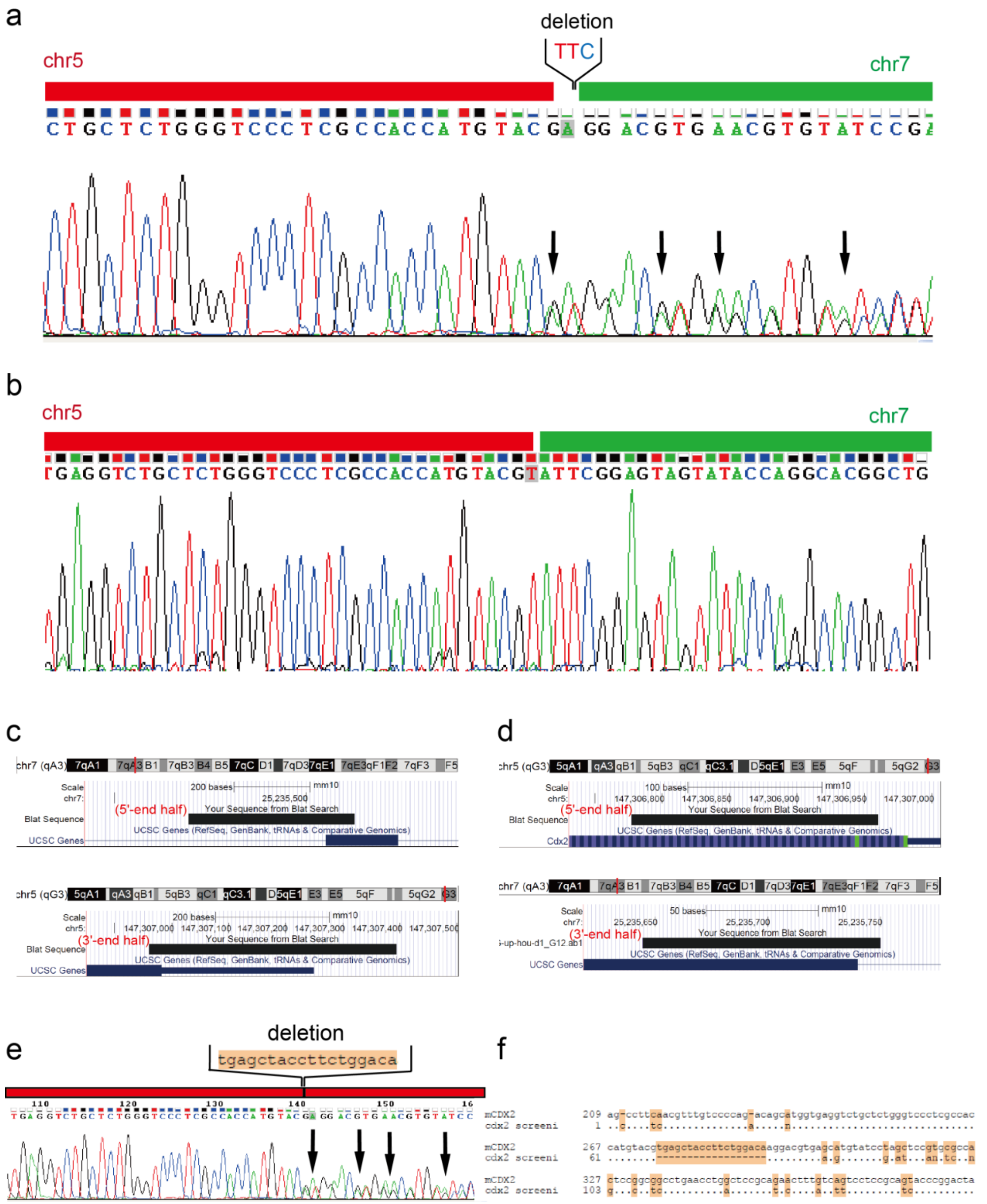

Figure S1 Identification of T (5:7) translocation by PCR analysis and sequencing.

(a) Sequence of the PCR product of the predicted T (5:7) chromosome-short. 3 nucleotides were deleted at the junction point. Double peaks are highlighted with arrows. **(b)** Sequence of the PCR product (in another pMD18-T clone) of the predicted T (5:7) chromosome-short. **(c)** Blat results in the UCSC Genome Browser using the sequence of the PCR product of the predicted T (5:7) chromosome-short. A red line in the chromosome shows the location of the blat sequence. **(d)** Blat results in

the UCSC Genome Browser using the sequence of the PCR product of the predicted T (5:7) chromosome-long. **(e)** Sequence of the PCR product of the chromosome 5 at Cdx2 gene locus. The appearance of double peaks (arrows) straight after the sgRNA binding site, and the short fragment deletion at the predicted Cas9-cuting site suggested the generation of indel mutation in the untranslocated chromosomes 5. **(f)** Blast results using the sequence of the PCR product and CDX2 mRNA.

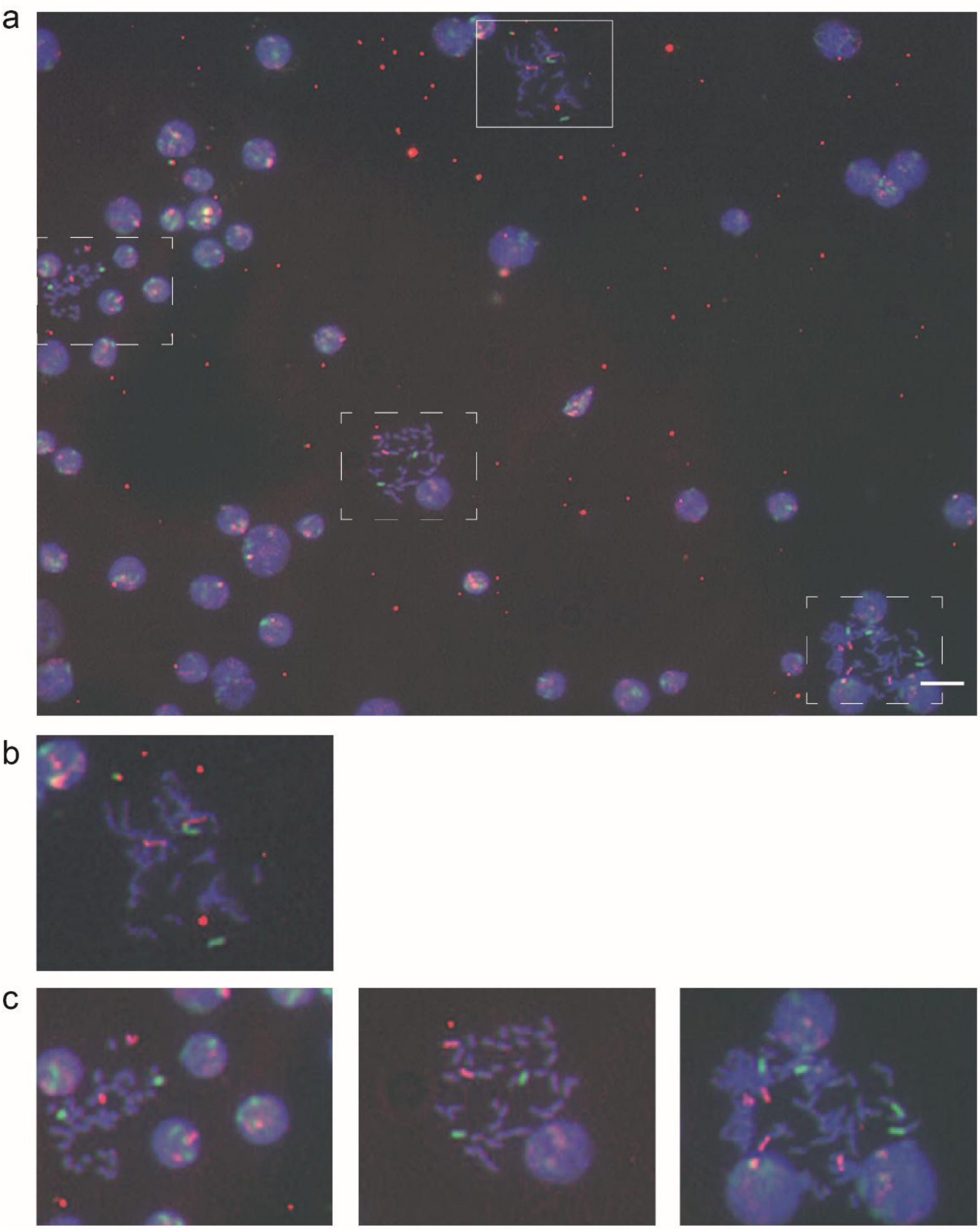

Figure S2| Detection of the T (5:7) translocation by chromosome painting in E14-cas9 cells infected with the two sgRNA lentiviral. **(a)**The pictures show chromosomes of 4 individual cells, one was detected T (5:7) translocation, and the other 3 cells did not show chromosomal translocation. **(b)** Zoomed in the cell carrying T (5:7) translocation（top frame in **a**）. **(c)** Zoomed in the cells with normal

chromosomes. Overall, we observed 100 cells whose chromosomes were clearly painted, and we detected 2 cells with the T (5:7) translocation. Scale bar, 10 µm.

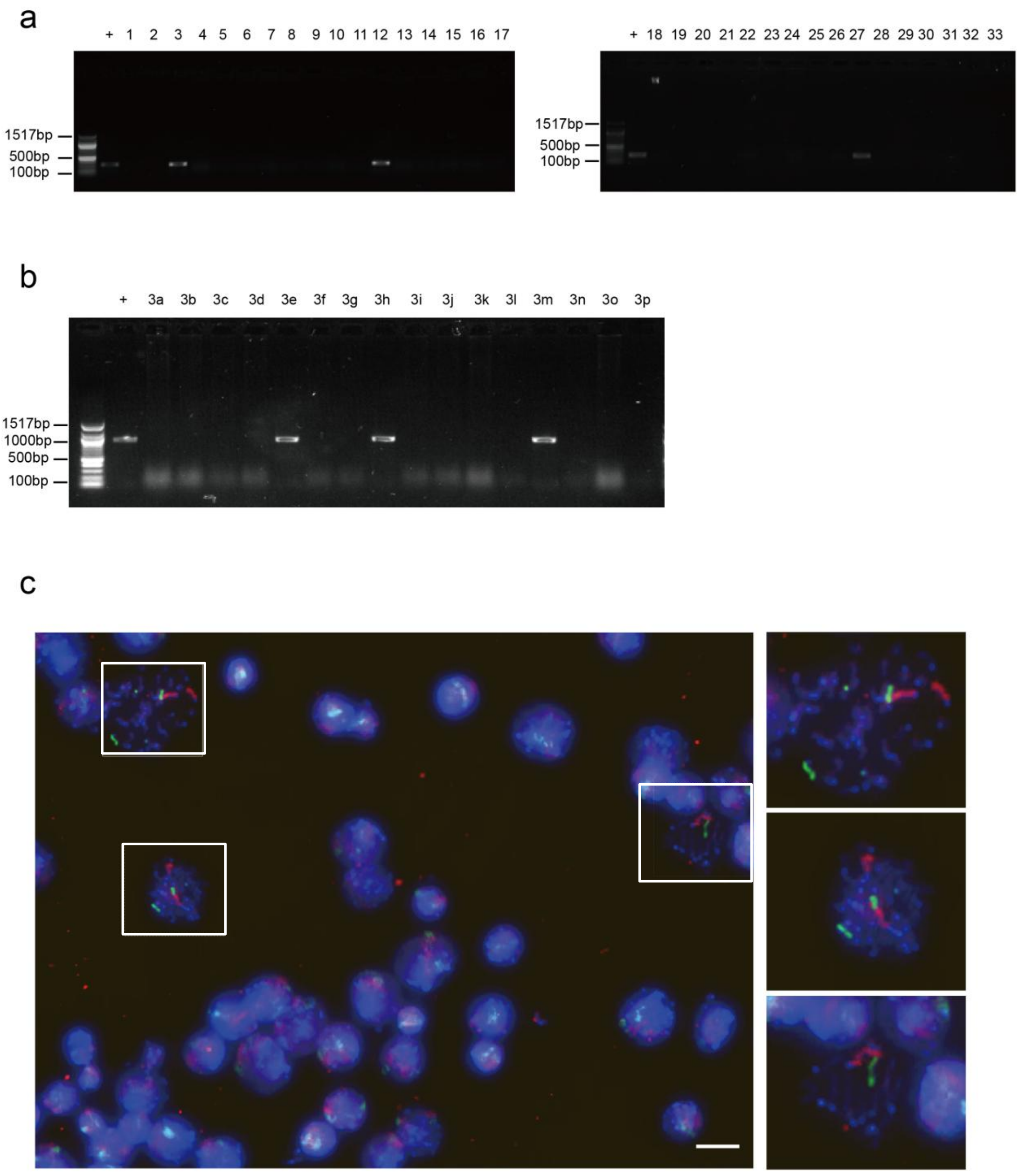

Figure S3| Establishment of mESC line carrying T (5:7) translocation. **(a)** T (5:7) chromosome-long was detected by PCR in 3 wells (No.3, No.12 and No.27) of the total 33 wells. **(b)** T (5:7) chromosome-short was detected by PCR in 3 (No.3e, No.3h and No.3m) of the total 16 colonies seeded from well No.3. **(c)**Every cells expanded from the colony No.3e carried the T (5:7) translocation. Chr5 was painted into red and Chr7 was painted into green. The inset is zoomed in on the chromosomes and the scale bar represents 10 µm.

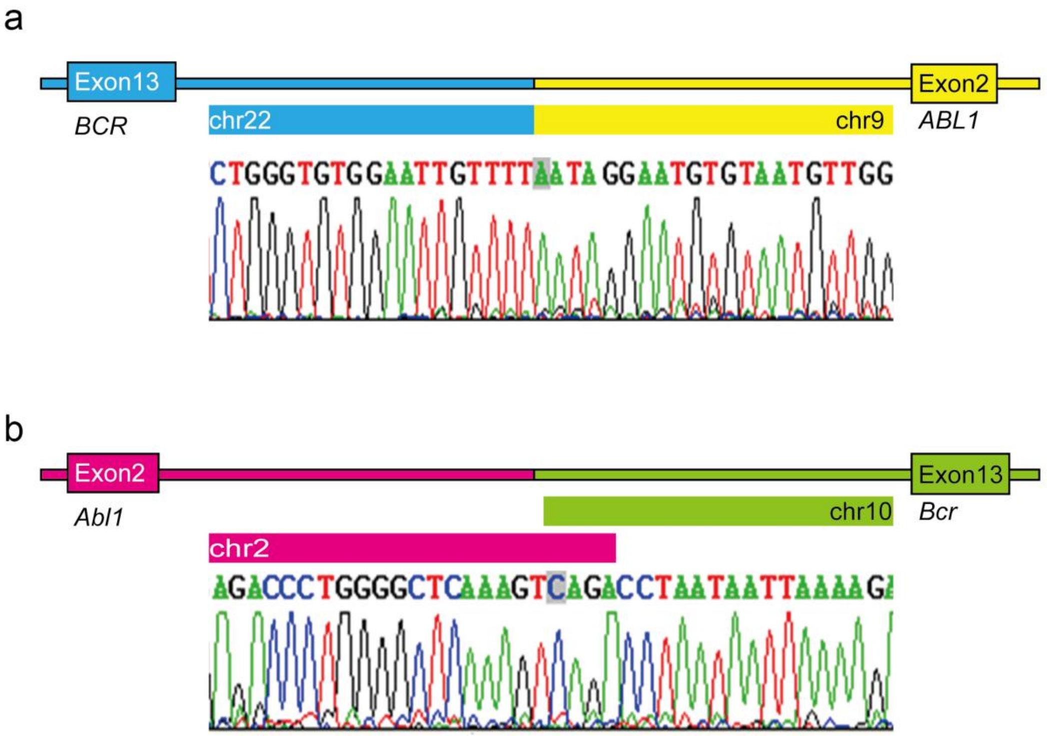

Figure S4| Induction of the BCR-ABL associated chromosomal translocation by the CRISPR/Cas9 system. (a) The PCR product sequence of the Philadelphia chromosome induced by co-expression of designed sgRNAs and Cas9 in HEK293 cells. The Philadelphia chromosome is a translocated chromosome related to human chronic myelogenous leukemia (CML). The result of the chromosomal translocation is that a fusion gene is created by juxtapositioning the *ABL1* gene on chromosome 9 to a part of the *BCR* gene on chromosome 22. **(b)** Partial sequence of the PCR product of the *Bcr-Abl1*fusion gene which was induced by co-expression of designed sgRNAs and Cas9 in mESCs. In mouse, the *Abl1* gene is located on chromosome 2 and the *Bcr* gene is located on chromosome 10.

Supplemental Table

Table 1. Chromosome translocation efficiency in E14 using CRISPR/Cass9

**Total Cells**	Heterozygous T(5:7)	homozygous T(5:7)	normal chromosome
**100**	2	0	98

Numbers of cells with T (5:7) translocation in a total 100 cells based on chromosome painting. 2 cells with heterozygous T (5:7) translocation were detected. No homozygous T (5:7) translocation was detected.

Table 2. Summary of E14-Cas9 with T (5:7) translocation clone pickup

	Cells origin	Cells/well	Wells	Wells with T (5:7)
**Step 1**	E14-Cas9 infected 2 sgRNAs	5	33	3
**Step 2**	Cells from 1 well with T (5:7) from step 1	1	16	3

Efficiency of E14-Cas9 with T (5:7) translocation through colony pick-up process. Two steps were taken for clone pick-up. In step 1, sgRNA-infected E14-Cas9 cells were passaged into 96-well plate at the density of approximately 5 cells per well. Out of 33 wells, the T (5:7) translocation was identified in 3 wells by PCR analysis. In step 2, cells from one translocation-positive well in step 1 were trypsinized and seeded into a new 96-well plate at the density of 1 cells per well. Out of 16 wells, the T (5:7) translocation was identified in 3 wells by PCR analysis.

Supplemental sequences

The target sequences of sgRNAs were:

Gsk3α-sgRNA target sequence (target chromosome7):
GAT TGGTAATGGC TCATTCGG

Cdx2-sgRNA target sequence (target chromosome7):
GTGAGCTACCTTCTGGACAAGG

hBCR-sgRNA target sequence (target human chromosome22):
GGTGTGGAATTGTTTTTCCCGG

hABL1-sgRNA target sequence (target human chromosome9):
AGATGTTAAGAAATGAAATAGG

mBCR-sgRNA target sequence (target mouse chromosome10):
AGGAGGCCTGGGCTACCCAAGG

mABL1-sgRNA target sequence (target mouse chromosome2):
TTTTACTTAAACCACCATAAGG

The protospacer-associated motif (PAM) sequences were underlined.

Primer sequences used in real-time PCR experiments：

Gene	Forward sequence(5’-3’)	Reverse sequence(5’-3’)
Gapdh	TGTGAGGGAGATGCTCAGTG	TGTTCCTACCCCCAATGTGT
Oct4	AAGCAGAAGAGGATCACCTTG	TTCTTAAGGCTGAGCTGCAAG
Nanog	TCCAGAAGAGGGCGTCAGAT	CAAATCCCAGCAACCACATG
Sox1	CTCCTCGGCTGAATTCTTTG	TGTAATCCGGGTGTTCCTTC
Nestin	CTCGAGCAGGAAGTGGTAGG	TTGGGACCAGGGACTGTTAG
Brachyury	CCGGTGCTGAAGGTAAATGT	CCTCCATTGAGCTTGTTGGT
Mixl1	TTGAATTGAACCCTGTTGTCCC	GAAACCCGTTCTCCCATCCACC
Afp	TCGTATTCCAACAGGAGG	AGGCTTTTGCTTCACCAG
Foxa2	CCTCAAGGGAGCAGTCTCAC	TTTCTCCTGGTCCGGTACAC

Sequence of PCR product using primer chr-long-p1and chr-long-p2：

**5’-ACGCGGNGGGCCAGGATGGCCCTGGGGACTGAGCGCTGTCCAAGT
TCGCCGTAGCAGCCGCCGCGGCCGCCACGTGGTAACCACCGTAGTCC
GGGTACTGCGGAGGACTGACAAAGTTCTGCGGAGCCAGGTTCAGGCC
GCCGGAGTGGCGCACGGAGCTAGGATACATGCTCACGTCCTTGT*AGC*
*TG*TACCAATCACTTTGATGTCAGTGTAAGCCACTTCTTGGGAACGCTC
TGGGCCTTGGCCTACAGTGGCTACCACTGTGGTCACCTTCCCGCTGT
CACCTAAGGAACA-3’**

Sequence of PCR product using primer chr-short-p1 and chr-short-p2：

**5’-TATTCCCCCCTCCCAATCATTCTCTTCCTCCCCCCTCTCCTTGGGCC
CATGCTTAAGTTCCAATGAAATCCCAGGCTCAAGCTGGTGAGCTGCT
ATGGTATATAAATGTGCTTGCCATGCGAGCTTCAAGTCCTGAGTTCAG
TCTCTGAATTCCACAGTGGAAGGTGAACCAACTTCCAACAGCTGCTCC
TGACCTCCACAGACAGACCATGGCCACACTTACAGACAGACATAACG
TAGGCACACATTAATAAGTAATATTGAGAACATGAAATTCCGGGCTCC
AACTCTATTGCTAGGTACACTCAGCAGTCACTCCCAGCGCCCAAACCT
CCAGTCTACCCAAGCTACCTTGAACCTTTTGTCCTGAAGAACCTTCTT
GATGGCCACCAGTTCCCTCGTCTCTGCCAGCCGTGCCTGGTATACCA
GAAGGTAGCTCACGTACATGGTGGCGAGGGACCCAGAGCAGACCTCA
CCATGCTGTCTGGGGACAAACGTTGAAGGCTGCCAGAGGCGCGAGGC
GCAGGGGGCTAGAGATAAAGGTAGCTGGCTGCACCTCAGCCCACGGT**

**GCTCCGTCGGCTCCTTGCAGAGCTTCTGCCTCCCGGGCAGTCCCTCC
CTCCGTCCTGCCTCCTCCCCTCTCTTCCTTCTTTCCTCCCACCTCCTTG
CCAGTAAGCTGTAGAGGCGGGGATGACCCGCCGCAGGTTGGCTCGC
GGAGTCCCAGGCCAGCGGCCTTACGTGATTAACGAGTGTTTACAAGA
CTCTATTAGTAATGACACAGACACCAATGGCTGGAGACGTCGAGGCG
CAGCGCGCTACGCACACAGTCCCCACCACCACCACCCCCAGAAACAC-
3**

PCR product sequence of BCR/ABL1 translocation in mESCs：

**5’-TCTGTTGCTCAGGAGTACACAGGGGACAACTCTGCCTTGGCATCTA
AGCTCATACAAATTTACTCATCAAGAGTTTTGTTCCTATTGCAAGGAA
ACGTTTTAGGGAAAAATGCTTTTGCTTGGGGAATTAAAAACAGTAGAA
AACCCTCAAGTTTCCAGCCTGGGGTTCAGGTGTCTTCTGTAGGAACAT
GGGGAAACGTGTAGCTCTGGAGCCCCCAAGGGTGGATGCTATGCTCT
GCAGGCATTTCTGCTCTCAAATTATAAAGGTCTTGGGAGCAAAGGGC
AGGCCACTCAGAGCCACCACCCCCCCCCTTTGCTTGCCATCTCTCCTT
TACCTTTAGTGATGCTGAGAGTGTTATCTCCACTGGCCACAAAATCAT
AGAGTGCCACAAAAAGGTTGGGGTCATTTTCACTGGGCCCAGCAAGA
AGGTTTTCCTTGGAGTTCCATCGAGCTGCTTCGCTGAGACCCTGGGG
CTCAAAGTCAGACCTAATAATTAAAAGACCTGCCAGCAGGGGGCAGC
TCATGCCTGCAGGATCTGAGACAGATATCTGTGGGGGAGGGGTGGGA
TGGGGGCGGAGAANCANAGGAAAACAATCCCTGGCCTTCTTAGGGGA
CAATTCTTCTTAGTTAAAGTTTACTCACAGGGTCCATCACATCCCCTC
CTTGAGCTACTTACTTGAGCTCTGCTTAAAGCCGGTGGCTGA -3**

PCR product sequence of BCR/ABL1 translocation in HEK293T：

**CTTTGATCTCTTGCGCAGATGATGAGTCTCCGGGGCTCTATGGGTTTC
TGAATGTCATCGTCCACTCAGCCACTGGATTTAAGCAGAGTTCAAGTA
AGTACTGGTTTGGGGAGGAGGGTTGCAGCGGCCGAGCCAGGGTCTC
CACCCAGGAAGGACTAATCGGGCAGGGTGTGGGGAAACAGGGAGGT
TGTTCAGATGACCACGGGACACCTTTGACCCTGGCCGCTGTGGAGTG
TTTGTGCTGGTTGATGCCTTCTGGGTGTGGAATTGTTTTAATAGGAAT
GTGTAATGTTGGAAACACAAATATTTTTGCTTCTGAGAATAAAACTAA
TTTTTTCTCCCAATTTTCTCTTCCTTTTTCTTTTTTCTGTTCCCCCCTTT
CTCTTCCAGAAGCCCTTCAGCGGCCAGTAGCATCTGACTTTGAGCCTC
AGGGTCTGAGTGAAGCCGCTCGTTGGAACTCCAAGGAAAACCTTCTC
GCTGGACCCAGTGAAAATGACCCCAACCTTTTCGTTGCACTGTATGAT
TTTGTGGCCAGTGGAGATAACACTCTAAGCATAA**
